# {2-[Bis(2,4-di-*tert*-butyl­phen­oxy)phosphan­yloxy-κ*P*]-3,5-di-*tert*-butyl­phenyl-κ*C* ^1^}[(1,2,5,6-η)-cyclo­octa-1,5-diene]rhodium(I) toluene monosolvate

**DOI:** 10.1107/S1600536812002851

**Published:** 2012-01-31

**Authors:** Detlef Selent, Anke Spannenberg, Armin Börner

**Affiliations:** a Leibniz-Institut für Katalyse e.V. an der Universität Rostock, Albert-Einstein-Strasse 29a, 18059 Rostock, Germany

## Abstract

The reaction of (η^3^-all­yl)[(1,2,5,6-η)-cyclo­octa-1,5-diene]rhodium(I) with tris­(2,4-di-*tert*-butyl­phen­yl)phosphite in toluene produces the title compound, [Rh(C_42_H_62_O_3_P)(C_8_H_12_)]·C_7_H_8_, by spontaneous metallation at one of the nonsubstituted phenyl *ortho*-C atoms of the phosphite mol­ecule. The coordination geometry at the Rh^I^ ion is distorted square-planar. The toluene solvent mol­ecule is disordered over two different orientations, with site-occupation factors of 0.810 (2) and 0.190 (2).

## Related literature

For the structure of a phenyl ester of diisopropyl phosphinous acid which is *ortho*-metallated with rhodium, see: Ruhland *et al.* (2008 ▶). A series of pincer-type complexes exhibit a similar five-membered cyclic structural motif; see, for example: Rubio *et al.* (2007 ▶); Salem *et al.* (2006 ▶). The title compound represents a catalyst precursor for the catalytic olefin hydro­formyl­ation reaction; see: Selent *et al.* (2007 ▶).
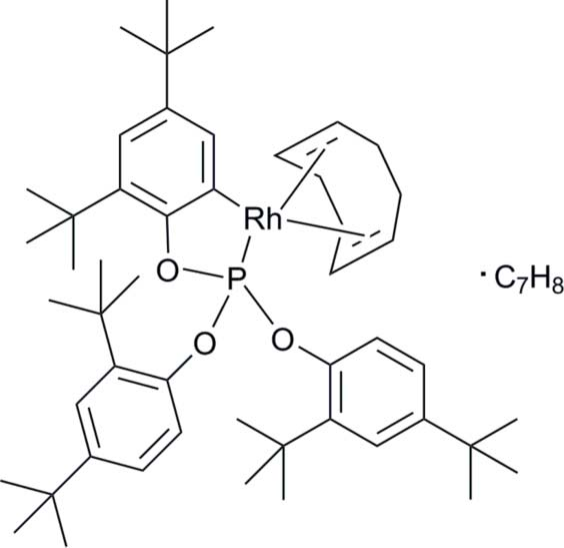


## Experimental

### 

#### Crystal data

[Rh(C_42_H_62_O_3_P)(C_8_H_12_)]·C_7_H_8_
*M* *_r_* = 949.11
Triclinic, 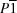

*a* = 11.1212 (3) Å
*b* = 12.5865 (3) Å
*c* = 20.0690 (5) Å
α = 106.891 (1)°
β = 102.344 (1)°
γ = 94.483 (1)°
*V* = 2596.13 (11) Å^3^
*Z* = 2
Mo *K*α radiation
μ = 0.40 mm^−1^
*T* = 150 K
0.44 × 0.33 × 0.09 mm

#### Data collection

Bruker Kappa APEXII DUO diffractometer
Absorption correction: multi-scan (*SADABS*; Bruker, 2008 ▶) *T*_min_ = 0.682, *T*_max_ = 0.746
106645 measured reflections
11927 independent reflections
11089 reflections with *I* > 2σ(*I*)
*R*_int_ = 0.027

#### Refinement

*R*[*F* ^2^ > 2σ(*F* ^2^)] = 0.027
*wR*(*F* ^2^) = 0.069
*S* = 1.02
11927 reflections
617 parameters
212 restraints
H-atom parameters constrained
Δρ_max_ = 0.97 e Å^−3^
Δρ_min_ = −0.61 e Å^−3^

### 

Data collection: *APEX2* (Bruker, 2011 ▶); cell refinement: *SAINT* (Bruker, 2009 ▶); data reduction: *SAINT*; program(s) used to solve structure: *SHELXS97* (Sheldrick, 2008 ▶); program(s) used to refine structure: *SHELXL97* (Sheldrick, 2008 ▶); molecular graphics: *XP* in *SHELXTL* (Sheldrick, 2008 ▶); software used to prepare material for publication: *SHELXTL*.

## Supplementary Material

Crystal structure: contains datablock(s) I, global. DOI: 10.1107/S1600536812002851/bt5795sup1.cif

Structure factors: contains datablock(s) I. DOI: 10.1107/S1600536812002851/bt5795Isup2.hkl

Additional supplementary materials:  crystallographic information; 3D view; checkCIF report

## Figures and Tables

**Fig. 1.**
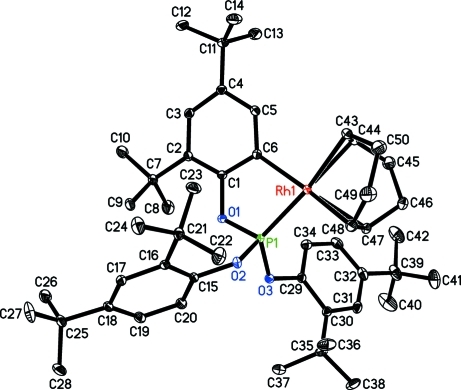
The molecular structure of the title compound showing the atom-labelling scheme. Hydrogen atoms and the disordered toluene are omitted for clarity. Displacement ellipsoids are drawn at the 30% probability level.

## supplementary crystallographic information

### Comment

The reaction of (η^3^-allyl)[(1,2,5,6-η)-cycloocta-1,5-diene]rhodium(I) with tri-(2,4-di-*tert.*-butylphenyl)-phosphite at room temperature affords the activation of one of the three *ortho* C—H bonds available at the phosphite phenyl groups. Subsequent Rh—C bond formation gives the title compound (figure 1). To the best of our knowledge, this is the first example for the direct formation of a rhodaoxaphospholane substructure using a π-allyl rhodium(I) complex as a precursor. In the title compound the coordination geometry at the rhodium centre is distorted square-planar. The Rh1—C6 distance 2.0771 (14) Å as well as the P1—Rh1—C6 angle 79.14 (4)° fit well to data from literature (Ruhland *et al.*, 2008). A more pronounced variation in the respective metal carbon distance has been found for pincer type complexes of Rh(I) and Rh(III) which do contain the same five-membered ring substructure (for example: Rubio *et al.*, 2007; Salem *et al.*, 2006). In the title compound one equivalent of toluene solvent is cocrystallized adopting two different orientations.

### Experimental

To a solution of (η^3^-allyl)[(1,2,5,6-η)-cycloocta-1,5-diene]rhodium(I) (0.552 g, 2.19 mmol) in pentane (15 ml) was added a solution of tri-(2,4-di-*tert.*-butylphenyl)-phosphite (1.416 g, 2.19 mmol) in toluene (15 ml) at room temperature. After stirring the mixture for 2 h, the solvent has been removed *in vacuo*. The residue was dissolved in toluene (12 ml) and stored at 5°C for three days to give a deep orange crystalline material. Yield: 1.36 g (65%) of the title compound, which did contain crystals suitable for X-ray analysis. ^31^P-NMR (400 MHz, CD_2_Cl_2_): 146.9 (d,^1^*J*_PRh_ = 317.9 Hz) p.p.m.. ^13^C-NMR (400 MHz, CD_2_Cl_2_): 149.07 (dd, ^1^*J*_CRh_ = 35.2 Hz, ^2^*J*_CP_ = 11.5 Hz) p.p.m..

### Refinement

H atoms were placed in idealized positions with d(C—H) = 0.95 Å (CH), 0.99 Å (CH_2_) and 0.98 Å (CH_3_) and refined using a riding model with *U*_iso_(H) fixed at 1.2 *U*_eq_(C) for CH, CH_2_ and 1.5 *U*_eq_(C) for CH_3_. AFIX 66 and DANG instructions were used to improve the geometry of the disordered toluene. Additionally, the anisotropic displacement parameters of C atoms of this solvent molecule were restrained to be equal (SIMU).

### Crystal data

**Table:**

[Rh(C_42_H_62_O_3_P)(C_8_H_12_)]·C_7_H_8_	*Z* = 2
*M**_r_* = 949.11	*F*(000) = 1016
Triclinic, *P*1	*D*_x_ = 1.214 Mg m^−^^3^
*a* = 11.1212 (3) Å	Mo *K*α radiation, λ = 0.71073 Å
*b* = 12.5865 (3) Å	Cell parameters from 9854 reflections
*c* = 20.0690 (5) Å	θ = 2.4–28.8°
α = 106.891 (1)°	µ = 0.40 mm^−^^1^
β = 102.344 (1)°	*T* = 150 K
γ = 94.483 (1)°	Plate, orange
*V* = 2596.13 (11) Å^3^	0.44 × 0.33 × 0.09 mm

### Data collection

**Table:**

Bruker Kappa APEXII DUO diffractometer	11927 independent reflections
Radiation source: fine-focus sealed tube	11089 reflections with *I* > 2σ(*I*)
curved graphite	*R*_int_ = 0.027
ω and φ scans	θ_max_ = 27.5°, θ_min_ = 1.7°
Absorption correction: multi-scan (*SADABS*; Bruker, 2008)	*h* = −14→14
*T*_min_ = 0.682, *T*_max_ = 0.746	*k* = −16→16
106645 measured reflections	*l* = −26→26

### Refinement

**Table:**

Refinement on *F*^2^	Primary atom site location: structure-invariant direct methods
Least-squares matrix: full	Secondary atom site location: difference Fourier map
*R*[*F*^2^ > 2σ(*F*^2^)] = 0.027	Hydrogen site location: inferred from neighbouring sites
*wR*(*F*^2^) = 0.069	H-atom parameters constrained
*S* = 1.02	*w* = 1/[σ^2^(*F*_o_^2^) + (0.0301*P*)^2^ + 2.2712*P*] where *P* = (*F*_o_^2^ + 2*F*_c_^2^)/3
11927 reflections	(Δ/σ)_max_ = 0.001
617 parameters	Δρ_max_ = 0.97 e Å^−^^3^
212 restraints	Δρ_min_ = −0.61 e Å^−^^3^

### Special details

**Table:**

Geometry. All e.s.d.'s (except the e.s.d. in the dihedral angle between two l.s. planes) are estimated using the full covariance matrix. The cell e.s.d.'s are taken into account individually in the estimation of e.s.d.'s in distances, angles and torsion angles; correlations between e.s.d.'s in cell parameters are only used when they are defined by crystal symmetry. An approximate (isotropic) treatment of cell e.s.d.'s is used for estimating e.s.d.'s involving l.s. planes.
Refinement. Refinement of *F*^2^ against ALL reflections. The weighted *R*-factor *wR* and goodness of fit *S* are based on *F*^2^, conventional *R*-factors *R* are based on *F*, with *F* set to zero for negative *F*^2^. The threshold expression of *F*^2^ > σ(*F*^2^) is used only for calculating *R*-factors(gt) *etc*. and is not relevant to the choice of reflections for refinement. *R*-factors based on *F*^2^ are statistically about twice as large as those based on *F*, and *R*- factors based on ALL data will be even larger.

### Fractional atomic coordinates and isotropic or equivalent isotropic displacement parameters (Å^2^)

**Table:**

	*x*	*y*	*z*	*U*_iso_*/*U*_eq_	Occ. (<1)
C1	0.13413 (14)	0.38493 (12)	0.22389 (8)	0.0159 (3)	
C2	0.00968 (13)	0.33864 (12)	0.21048 (8)	0.0164 (3)	
C3	−0.02045 (14)	0.29798 (13)	0.26393 (8)	0.0180 (3)	
H3	−0.1040	0.2654	0.2571	0.022*	
C4	0.06639 (14)	0.30314 (12)	0.32650 (8)	0.0177 (3)	
C5	0.18872 (14)	0.35135 (13)	0.33572 (8)	0.0187 (3)	
H5	0.2485	0.3554	0.3783	0.022*	
C6	0.22786 (14)	0.39405 (12)	0.28544 (8)	0.0171 (3)	
C7	−0.08877 (14)	0.33156 (13)	0.14213 (8)	0.0201 (3)	
C8	−0.09912 (18)	0.44891 (16)	0.13496 (11)	0.0331 (4)	
H8A	−0.0189	0.4824	0.1317	0.050*	
H8B	−0.1219	0.4965	0.1771	0.050*	
H8C	−0.1632	0.4427	0.0915	0.050*	
C9	−0.05480 (15)	0.25577 (15)	0.07586 (9)	0.0262 (3)	
H9A	−0.0495	0.1808	0.0805	0.039*	
H9B	0.0257	0.2879	0.0722	0.039*	
H9C	−0.1189	0.2502	0.0326	0.039*	
C10	−0.21805 (15)	0.28027 (18)	0.14265 (10)	0.0328 (4)	
H10A	−0.2781	0.2769	0.0983	0.049*	
H10B	−0.2436	0.3269	0.1841	0.049*	
H10C	−0.2151	0.2043	0.1459	0.049*	
C11	0.03018 (15)	0.26319 (13)	0.38602 (8)	0.0206 (3)	
C12	−0.10222 (16)	0.19816 (15)	0.36252 (10)	0.0272 (3)	
H12A	−0.1096	0.1333	0.3198	0.041*	
H12B	−0.1621	0.2474	0.3512	0.041*	
H12C	−0.1196	0.1722	0.4015	0.041*	
C13	0.0375 (2)	0.36651 (16)	0.45093 (10)	0.0351 (4)	
H13A	0.0163	0.3424	0.4898	0.053*	
H13B	−0.0213	0.4150	0.4370	0.053*	
H13C	0.1222	0.4082	0.4675	0.053*	
C14	0.11906 (17)	0.18553 (17)	0.40839 (11)	0.0329 (4)	
H14A	0.0951	0.1615	0.4466	0.049*	
H14B	0.2044	0.2259	0.4260	0.049*	
H14C	0.1145	0.1195	0.3669	0.049*	
C15	0.26902 (13)	0.31172 (12)	0.05287 (7)	0.0158 (3)	
C16	0.25632 (13)	0.19485 (12)	0.02820 (8)	0.0151 (3)	
C17	0.17166 (14)	0.14197 (12)	−0.03858 (8)	0.0172 (3)	
H17	0.1600	0.0623	−0.0568	0.021*	
C18	0.10366 (14)	0.19934 (13)	−0.07977 (8)	0.0186 (3)	
C19	0.12243 (15)	0.31595 (14)	−0.05279 (8)	0.0222 (3)	
H19	0.0782	0.3577	−0.0797	0.027*	
C20	0.20485 (15)	0.37182 (13)	0.01283 (8)	0.0211 (3)	
H20	0.2175	0.4516	0.0305	0.025*	
C21	0.32860 (14)	0.12665 (13)	0.07109 (8)	0.0193 (3)	
C22	0.46945 (16)	0.16248 (16)	0.08532 (11)	0.0317 (4)	
H22A	0.4915	0.2419	0.1142	0.048*	
H22B	0.4921	0.1514	0.0394	0.048*	
H22C	0.5145	0.1169	0.1114	0.048*	
C23	0.29187 (18)	0.14320 (16)	0.14245 (9)	0.0298 (4)	
H23A	0.2009	0.1322	0.1335	0.045*	
H23B	0.3258	0.2194	0.1747	0.045*	
H23C	0.3254	0.0885	0.1647	0.045*	
C24	0.30048 (19)	0.00093 (14)	0.02933 (10)	0.0322 (4)	
H24A	0.3489	−0.0402	0.0574	0.048*	
H24B	0.3231	−0.0121	−0.0167	0.048*	
H24C	0.2115	−0.0254	0.0205	0.048*	
C25	0.00794 (15)	0.13847 (14)	−0.15105 (8)	0.0231 (3)	
C26	−0.12261 (16)	0.15377 (17)	−0.13958 (10)	0.0311 (4)	
H26A	−0.1852	0.1156	−0.1847	0.047*	
H26B	−0.1292	0.2340	−0.1243	0.047*	
H26C	−0.1367	0.1215	−0.1025	0.047*	
C27	0.0149 (2)	0.01295 (17)	−0.17757 (11)	0.0410 (5)	
H27A	0.0988	0.0021	−0.1834	0.062*	
H27B	−0.0458	−0.0219	−0.2239	0.062*	
H27C	−0.0039	−0.0219	−0.1425	0.062*	
C28	0.02878 (19)	0.1894 (2)	−0.20926 (10)	0.0390 (5)	
H28A	0.1135	0.1836	−0.2151	0.059*	
H28B	0.0174	0.2685	−0.1950	0.059*	
H28C	−0.0313	0.1485	−0.2549	0.059*	
C29	0.35089 (13)	0.67593 (12)	0.20433 (8)	0.0163 (3)	
C30	0.43783 (14)	0.75291 (13)	0.19409 (8)	0.0185 (3)	
C31	0.46435 (15)	0.85918 (13)	0.24577 (9)	0.0243 (3)	
H31	0.5227	0.9140	0.2409	0.029*	
C32	0.41048 (17)	0.89007 (13)	0.30418 (9)	0.0257 (3)	
C33	0.32305 (17)	0.81119 (14)	0.30994 (9)	0.0254 (3)	
H33	0.2834	0.8301	0.3484	0.031*	
C34	0.29276 (15)	0.70453 (13)	0.25988 (8)	0.0210 (3)	
H34	0.2318	0.6511	0.2639	0.025*	
C35	0.49977 (15)	0.72371 (15)	0.13084 (9)	0.0246 (3)	
C36	0.57819 (19)	0.62909 (18)	0.13393 (12)	0.0380 (5)	
H36A	0.6400	0.6514	0.1802	0.057*	
H36B	0.6208	0.6151	0.0949	0.057*	
H36C	0.5237	0.5605	0.1286	0.057*	
C37	0.39979 (18)	0.68896 (17)	0.05927 (9)	0.0321 (4)	
H37A	0.3437	0.6224	0.0557	0.048*	
H37B	0.4400	0.6717	0.0192	0.048*	
H37C	0.3520	0.7507	0.0574	0.048*	
C38	0.58701 (18)	0.82573 (18)	0.13219 (12)	0.0385 (5)	
H38A	0.5395	0.8875	0.1299	0.058*	
H38B	0.6238	0.8052	0.0908	0.058*	
H38C	0.6533	0.8495	0.1767	0.058*	
C39	0.4469 (2)	1.00861 (15)	0.35864 (10)	0.0393 (5)	
C40	0.4058 (3)	1.09418 (19)	0.32098 (14)	0.0680 (9)	
H40A	0.4208	1.1691	0.3566	0.102*	
H40B	0.3169	1.0738	0.2971	0.102*	
H40C	0.4534	1.0944	0.2852	0.102*	
C41	0.5863 (3)	1.0308 (2)	0.39091 (14)	0.0617 (7)	
H41A	0.6097	0.9751	0.4148	0.093*	
H41B	0.6095	1.1063	0.4260	0.093*	
H41C	0.6299	1.0254	0.3527	0.093*	
C42	0.3819 (3)	1.02265 (18)	0.42067 (11)	0.0488 (6)	
H42A	0.4039	0.9672	0.4450	0.073*	
H42B	0.2915	1.0113	0.4014	0.073*	
H42C	0.4091	1.0984	0.4550	0.073*	
C43	0.49889 (15)	0.42810 (15)	0.39537 (9)	0.0244 (3)	
H43	0.4424	0.3598	0.3779	0.029*	
C44	0.45110 (16)	0.52555 (14)	0.41499 (8)	0.0243 (3)	
H44	0.3649	0.5186	0.4138	0.029*	
C45	0.5204 (2)	0.64357 (16)	0.43842 (10)	0.0353 (4)	
H45A	0.5633	0.6648	0.4900	0.042*	
H45B	0.4592	0.6955	0.4332	0.042*	
C46	0.61630 (18)	0.65943 (16)	0.39648 (10)	0.0330 (4)	
H46A	0.6322	0.7399	0.4007	0.040*	
H46B	0.6955	0.6384	0.4186	0.040*	
C47	0.57685 (14)	0.59097 (14)	0.31753 (9)	0.0236 (3)	
H47	0.5300	0.6241	0.2856	0.028*	
C48	0.60325 (15)	0.48473 (16)	0.28815 (9)	0.0262 (3)	
H48	0.5770	0.4532	0.2373	0.031*	
C49	0.66954 (18)	0.41334 (19)	0.32842 (12)	0.0365 (4)	
H49A	0.7604	0.4367	0.3386	0.044*	
H49B	0.6503	0.3341	0.2971	0.044*	
C50	0.63515 (17)	0.42021 (18)	0.39924 (11)	0.0358 (4)	
H50A	0.6556	0.3528	0.4127	0.043*	
H50B	0.6862	0.4868	0.4375	0.043*	
O1	0.16873 (10)	0.42629 (9)	0.17109 (6)	0.0179 (2)	
O2	0.35110 (10)	0.36957 (9)	0.12007 (5)	0.0177 (2)	
O3	0.32268 (10)	0.56650 (9)	0.15626 (5)	0.0181 (2)	
P1	0.31701 (3)	0.45944 (3)	0.185496 (19)	0.01476 (7)	
Rh1	0.407621 (10)	0.466102 (10)	0.294657 (6)	0.01623 (4)	
C51A	1.04794 (16)	0.19961 (13)	0.61315 (10)	0.0416 (5)	0.810 (2)
C52A	0.93112 (18)	0.15976 (11)	0.56597 (11)	0.0483 (5)	0.810 (2)
H52A	0.9106	0.0829	0.5376	0.058*	0.810 (2)
C53A	0.84431 (14)	0.23239 (16)	0.56033 (10)	0.0527 (5)	0.810 (2)
H53A	0.7645	0.2052	0.5281	0.063*	0.810 (2)
C54A	0.87432 (16)	0.34488 (15)	0.60186 (10)	0.0474 (5)	0.810 (2)
H54A	0.8150	0.3945	0.5980	0.057*	0.810 (2)
C55A	0.99114 (18)	0.38473 (11)	0.64904 (11)	0.0428 (5)	0.810 (2)
H55A	1.0116	0.4616	0.6774	0.051*	0.810 (2)
C56A	1.07795 (14)	0.31209 (14)	0.65469 (10)	0.0405 (5)	0.810 (2)
H56A	1.1578	0.3393	0.6869	0.049*	0.810 (2)
C57A	1.1426 (4)	0.1217 (3)	0.6204 (2)	0.0600 (7)	0.810 (2)
H57A	1.2190	0.1639	0.6557	0.090*	0.810 (2)
H57B	1.1611	0.0888	0.5738	0.090*	0.810 (2)
H57C	1.1091	0.0617	0.6364	0.090*	0.810 (2)
C51B	0.9051 (6)	0.2807 (5)	0.5938 (4)	0.0462 (6)	0.190 (2)
C52B	0.9225 (7)	0.1697 (5)	0.5667 (4)	0.0472 (7)	0.190 (2)
H52B	0.8583	0.1172	0.5304	0.057*	0.190 (2)
C53B	1.0339 (7)	0.1355 (5)	0.5929 (4)	0.0471 (8)	0.190 (2)
H53B	1.0458	0.0596	0.5745	0.057*	0.190 (2)
C54B	1.1278 (6)	0.2123 (6)	0.6462 (4)	0.0444 (8)	0.190 (2)
H54B	1.2039	0.1889	0.6640	0.053*	0.190 (2)
C55B	1.1104 (7)	0.3233 (6)	0.6732 (4)	0.0438 (7)	0.190 (2)
H55B	1.1746	0.3758	0.7096	0.053*	0.190 (2)
C56B	0.9990 (7)	0.3576 (5)	0.6470 (5)	0.0439 (7)	0.190 (2)
H56B	0.9871	0.4334	0.6655	0.053*	0.190 (2)
C57B	0.7868 (7)	0.3182 (8)	0.5641 (6)	0.0496 (14)	0.190 (2)
H57D	0.7903	0.3986	0.5884	0.074*	0.190 (2)
H57E	0.7164	0.2758	0.5719	0.074*	0.190 (2)
H57F	0.7762	0.3050	0.5124	0.074*	0.190 (2)

### Atomic displacement parameters (Å^2^)

**Table:**

	*U*^11^	*U*^22^	*U*^33^	*U*^12^	*U*^13^	*U*^23^
C1	0.0181 (7)	0.0161 (6)	0.0141 (6)	0.0008 (5)	0.0053 (5)	0.0051 (5)
C2	0.0153 (6)	0.0177 (7)	0.0146 (6)	0.0020 (5)	0.0031 (5)	0.0035 (5)
C3	0.0154 (7)	0.0196 (7)	0.0188 (7)	0.0006 (5)	0.0057 (6)	0.0052 (6)
C4	0.0199 (7)	0.0176 (7)	0.0166 (7)	0.0017 (5)	0.0071 (6)	0.0052 (5)
C5	0.0177 (7)	0.0227 (7)	0.0148 (7)	0.0008 (6)	0.0022 (5)	0.0065 (6)
C6	0.0160 (6)	0.0182 (7)	0.0155 (7)	−0.0004 (5)	0.0035 (5)	0.0041 (5)
C7	0.0149 (7)	0.0261 (8)	0.0177 (7)	0.0002 (6)	0.0013 (6)	0.0070 (6)
C8	0.0288 (9)	0.0327 (9)	0.0353 (10)	0.0073 (7)	−0.0036 (7)	0.0147 (8)
C9	0.0217 (8)	0.0334 (9)	0.0170 (7)	−0.0036 (7)	0.0019 (6)	0.0023 (6)
C10	0.0156 (7)	0.0560 (12)	0.0246 (8)	−0.0024 (7)	0.0010 (6)	0.0145 (8)
C11	0.0211 (7)	0.0241 (7)	0.0190 (7)	0.0012 (6)	0.0076 (6)	0.0092 (6)
C12	0.0234 (8)	0.0333 (9)	0.0293 (9)	−0.0013 (7)	0.0094 (7)	0.0159 (7)
C13	0.0470 (11)	0.0351 (10)	0.0220 (8)	−0.0042 (8)	0.0163 (8)	0.0044 (7)
C14	0.0297 (9)	0.0430 (11)	0.0369 (10)	0.0078 (8)	0.0098 (8)	0.0271 (9)
C15	0.0153 (6)	0.0181 (7)	0.0120 (6)	−0.0005 (5)	0.0027 (5)	0.0031 (5)
C16	0.0137 (6)	0.0175 (7)	0.0148 (6)	0.0017 (5)	0.0051 (5)	0.0054 (5)
C17	0.0172 (7)	0.0175 (7)	0.0154 (7)	0.0003 (5)	0.0045 (5)	0.0032 (5)
C18	0.0171 (7)	0.0240 (7)	0.0130 (6)	0.0005 (6)	0.0035 (5)	0.0043 (6)
C19	0.0253 (8)	0.0241 (8)	0.0176 (7)	0.0035 (6)	0.0018 (6)	0.0100 (6)
C20	0.0273 (8)	0.0164 (7)	0.0187 (7)	0.0013 (6)	0.0038 (6)	0.0060 (6)
C21	0.0182 (7)	0.0177 (7)	0.0199 (7)	0.0023 (5)	0.0007 (6)	0.0059 (6)
C22	0.0182 (8)	0.0321 (9)	0.0426 (10)	0.0067 (7)	0.0016 (7)	0.0119 (8)
C23	0.0393 (10)	0.0309 (9)	0.0232 (8)	0.0058 (7)	0.0066 (7)	0.0152 (7)
C24	0.0402 (10)	0.0182 (8)	0.0315 (9)	0.0065 (7)	−0.0037 (8)	0.0061 (7)
C25	0.0213 (7)	0.0293 (8)	0.0142 (7)	0.0002 (6)	−0.0002 (6)	0.0042 (6)
C26	0.0207 (8)	0.0441 (10)	0.0242 (8)	−0.0002 (7)	0.0001 (6)	0.0097 (8)
C27	0.0420 (11)	0.0338 (10)	0.0269 (9)	0.0047 (8)	−0.0107 (8)	−0.0069 (8)
C28	0.0353 (10)	0.0619 (13)	0.0162 (8)	−0.0062 (9)	0.0002 (7)	0.0147 (8)
C29	0.0166 (6)	0.0144 (6)	0.0162 (7)	0.0008 (5)	0.0017 (5)	0.0045 (5)
C30	0.0152 (7)	0.0207 (7)	0.0204 (7)	0.0012 (5)	0.0022 (5)	0.0097 (6)
C31	0.0225 (8)	0.0193 (7)	0.0278 (8)	−0.0031 (6)	−0.0024 (6)	0.0105 (6)
C32	0.0328 (9)	0.0176 (7)	0.0201 (7)	0.0051 (6)	−0.0042 (6)	0.0032 (6)
C33	0.0326 (9)	0.0256 (8)	0.0178 (7)	0.0102 (7)	0.0062 (6)	0.0049 (6)
C34	0.0211 (7)	0.0224 (7)	0.0213 (7)	0.0039 (6)	0.0068 (6)	0.0082 (6)
C35	0.0228 (8)	0.0304 (8)	0.0265 (8)	0.0021 (6)	0.0109 (6)	0.0150 (7)
C36	0.0369 (10)	0.0495 (12)	0.0455 (11)	0.0196 (9)	0.0272 (9)	0.0264 (10)
C37	0.0376 (10)	0.0382 (10)	0.0231 (8)	−0.0010 (8)	0.0098 (7)	0.0135 (7)
C38	0.0294 (9)	0.0487 (12)	0.0449 (11)	−0.0060 (8)	0.0131 (8)	0.0263 (10)
C39	0.0597 (13)	0.0196 (8)	0.0257 (9)	0.0037 (8)	−0.0059 (9)	0.0000 (7)
C40	0.131 (3)	0.0226 (10)	0.0397 (13)	0.0151 (13)	0.0024 (15)	0.0066 (9)
C41	0.0617 (16)	0.0530 (15)	0.0421 (13)	−0.0174 (12)	−0.0083 (12)	−0.0059 (11)
C42	0.0755 (16)	0.0330 (11)	0.0281 (10)	0.0189 (11)	0.0041 (10)	−0.0020 (8)
C43	0.0217 (8)	0.0311 (8)	0.0204 (7)	−0.0021 (6)	−0.0005 (6)	0.0139 (7)
C44	0.0280 (8)	0.0273 (8)	0.0131 (7)	−0.0059 (6)	0.0018 (6)	0.0045 (6)
C45	0.0468 (11)	0.0282 (9)	0.0225 (8)	−0.0097 (8)	0.0077 (8)	−0.0001 (7)
C46	0.0350 (10)	0.0302 (9)	0.0255 (9)	−0.0140 (7)	−0.0006 (7)	0.0068 (7)
C47	0.0160 (7)	0.0318 (8)	0.0219 (8)	−0.0058 (6)	−0.0004 (6)	0.0130 (7)
C48	0.0166 (7)	0.0390 (9)	0.0242 (8)	0.0010 (6)	0.0051 (6)	0.0128 (7)
C49	0.0252 (9)	0.0477 (11)	0.0456 (11)	0.0141 (8)	0.0122 (8)	0.0235 (9)
C50	0.0247 (9)	0.0469 (11)	0.0429 (11)	0.0071 (8)	0.0026 (8)	0.0284 (9)
O1	0.0157 (5)	0.0231 (5)	0.0153 (5)	−0.0021 (4)	0.0021 (4)	0.0091 (4)
O2	0.0175 (5)	0.0170 (5)	0.0144 (5)	0.0000 (4)	0.0006 (4)	0.0016 (4)
O3	0.0244 (5)	0.0143 (5)	0.0144 (5)	−0.0021 (4)	0.0045 (4)	0.0040 (4)
P1	0.01523 (17)	0.01513 (17)	0.01229 (16)	−0.00175 (13)	0.00200 (13)	0.00392 (13)
Rh1	0.01411 (6)	0.02073 (6)	0.01209 (6)	−0.00188 (4)	0.00111 (4)	0.00523 (4)
C51A	0.0544 (10)	0.0381 (9)	0.0384 (9)	0.0002 (8)	0.0269 (8)	0.0120 (8)
C52A	0.0590 (10)	0.0453 (9)	0.0390 (9)	−0.0075 (8)	0.0245 (8)	0.0059 (8)
C53A	0.0546 (11)	0.0594 (11)	0.0407 (10)	−0.0066 (9)	0.0183 (9)	0.0097 (9)
C54A	0.0520 (10)	0.0532 (10)	0.0413 (9)	0.0076 (9)	0.0212 (8)	0.0146 (9)
C55A	0.0542 (10)	0.0404 (9)	0.0375 (9)	0.0030 (8)	0.0230 (8)	0.0107 (8)
C56A	0.0509 (10)	0.0384 (9)	0.0358 (9)	−0.0003 (8)	0.0214 (8)	0.0115 (8)
C57A	0.0763 (17)	0.0483 (14)	0.0689 (16)	0.0169 (13)	0.0366 (14)	0.0232 (12)
C51B	0.0554 (11)	0.0468 (11)	0.0383 (10)	−0.0021 (10)	0.0232 (10)	0.0102 (10)
C52B	0.0572 (12)	0.0458 (11)	0.0391 (11)	−0.0046 (11)	0.0243 (11)	0.0084 (11)
C53B	0.0588 (13)	0.0442 (12)	0.0411 (12)	−0.0021 (12)	0.0262 (12)	0.0102 (12)
C54B	0.0564 (13)	0.0415 (12)	0.0406 (12)	0.0010 (12)	0.0258 (12)	0.0124 (12)
C55B	0.0550 (12)	0.0411 (11)	0.0393 (11)	0.0011 (11)	0.0242 (11)	0.0114 (11)
C56B	0.0538 (12)	0.0435 (12)	0.0381 (11)	0.0011 (11)	0.0229 (11)	0.0116 (11)
C57B	0.056 (2)	0.053 (2)	0.039 (2)	−0.003 (2)	0.019 (2)	0.011 (2)

### Geometric parameters (Å, °)

**Table:**

C1—C2	1.397 (2)	C34—H34	0.9500
C1—C6	1.405 (2)	C35—C38	1.538 (2)
C1—O1	1.4160 (17)	C35—C36	1.538 (3)
C2—C3	1.404 (2)	C35—C37	1.538 (2)
C2—C7	1.536 (2)	C36—H36A	0.9800
C3—C4	1.392 (2)	C36—H36B	0.9800
C3—H3	0.9500	C36—H36C	0.9800
C4—C5	1.397 (2)	C37—H37A	0.9800
C4—C11	1.538 (2)	C37—H37B	0.9800
C5—C6	1.401 (2)	C37—H37C	0.9800
C5—H5	0.9500	C38—H38A	0.9800
C6—Rh1	2.0771 (14)	C38—H38B	0.9800
C7—C10	1.533 (2)	C38—H38C	0.9800
C7—C9	1.535 (2)	C39—C41	1.516 (3)
C7—C8	1.536 (2)	C39—C40	1.531 (3)
C8—H8A	0.9800	C39—C42	1.543 (3)
C8—H8B	0.9800	C40—H40A	0.9800
C8—H8C	0.9800	C40—H40B	0.9800
C9—H9A	0.9800	C40—H40C	0.9800
C9—H9B	0.9800	C41—H41A	0.9800
C9—H9C	0.9800	C41—H41B	0.9800
C10—H10A	0.9800	C41—H41C	0.9800
C10—H10B	0.9800	C42—H42A	0.9800
C10—H10C	0.9800	C42—H42B	0.9800
C11—C13	1.532 (2)	C42—H42C	0.9800
C11—C14	1.533 (2)	C43—C44	1.361 (3)
C11—C12	1.533 (2)	C43—C50	1.513 (2)
C12—H12A	0.9800	C43—Rh1	2.2595 (15)
C12—H12B	0.9800	C43—H43	0.9500
C12—H12C	0.9800	C44—C45	1.510 (2)
C13—H13A	0.9800	C44—Rh1	2.2405 (15)
C13—H13B	0.9800	C44—H44	0.9500
C13—H13C	0.9800	C45—C46	1.528 (3)
C14—H14A	0.9800	C45—H45A	0.9900
C14—H14B	0.9800	C45—H45B	0.9900
C14—H14C	0.9800	C46—C47	1.515 (2)
C15—C20	1.386 (2)	C46—H46A	0.9900
C15—C16	1.394 (2)	C46—H46B	0.9900
C15—O2	1.4076 (17)	C47—C48	1.378 (3)
C16—C17	1.404 (2)	C47—Rh1	2.2298 (15)
C16—C21	1.538 (2)	C47—H47	0.9500
C17—C18	1.393 (2)	C48—C49	1.508 (2)
C17—H17	0.9500	C48—Rh1	2.2057 (16)
C18—C19	1.390 (2)	C48—H48	0.9500
C18—C25	1.533 (2)	C49—C50	1.530 (3)
C19—C20	1.384 (2)	C49—H49A	0.9900
C19—H19	0.9500	C49—H49B	0.9900
C20—H20	0.9500	C50—H50A	0.9900
C21—C24	1.531 (2)	C50—H50B	0.9900
C21—C23	1.533 (2)	O1—P1	1.6111 (11)
C21—C22	1.537 (2)	O2—P1	1.6012 (11)
C22—H22A	0.9800	O3—P1	1.6205 (11)
C22—H22B	0.9800	P1—Rh1	2.1813 (4)
C22—H22C	0.9800	C51A—C52A	1.3900
C23—H23A	0.9800	C51A—C56A	1.3900
C23—H23B	0.9800	C51A—C57A	1.506 (4)
C23—H23C	0.9800	C52A—C53A	1.3900
C24—H24A	0.9800	C52A—H52A	0.9500
C24—H24B	0.9800	C53A—C54A	1.3900
C24—H24C	0.9800	C53A—H53A	0.9500
C25—C27	1.528 (3)	C54A—C55A	1.3900
C25—C28	1.535 (2)	C54A—H54A	0.9500
C25—C26	1.536 (2)	C55A—C56A	1.3900
C26—H26A	0.9800	C55A—H55A	0.9500
C26—H26B	0.9800	C56A—H56A	0.9500
C26—H26C	0.9800	C57A—H57A	0.9800
C27—H27A	0.9800	C57A—H57B	0.9800
C27—H27B	0.9800	C57A—H57C	0.9800
C27—H27C	0.9800	C51B—C52B	1.3900
C28—H28A	0.9800	C51B—C56B	1.3900
C28—H28B	0.9800	C51B—C57B	1.4947 (17)
C28—H28C	0.9800	C52B—C53B	1.3900
C29—C34	1.379 (2)	C52B—H52B	0.9500
C29—O3	1.3989 (17)	C53B—C54B	1.3900
C29—C30	1.404 (2)	C53B—H53B	0.9500
C30—C31	1.398 (2)	C54B—C55B	1.3900
C30—C35	1.537 (2)	C54B—H54B	0.9500
C31—C32	1.399 (3)	C55B—C56B	1.3900
C31—H31	0.9500	C55B—H55B	0.9500
C32—C33	1.381 (3)	C56B—H56B	0.9500
C32—C39	1.534 (2)	C57B—H57D	0.9800
C33—C34	1.388 (2)	C57B—H57E	0.9800
C33—H33	0.9500	C57B—H57F	0.9800
			
C2—C1—C6	125.46 (13)	C35—C36—H36C	109.5
C2—C1—O1	117.29 (12)	H36A—C36—H36C	109.5
C6—C1—O1	117.25 (12)	H36B—C36—H36C	109.5
C1—C2—C3	115.44 (13)	C35—C37—H37A	109.5
C1—C2—C7	123.01 (13)	C35—C37—H37B	109.5
C3—C2—C7	121.55 (13)	H37A—C37—H37B	109.5
C4—C3—C2	123.04 (14)	C35—C37—H37C	109.5
C4—C3—H3	118.5	H37A—C37—H37C	109.5
C2—C3—H3	118.5	H37B—C37—H37C	109.5
C3—C4—C5	117.72 (13)	C35—C38—H38A	109.5
C3—C4—C11	122.20 (13)	C35—C38—H38B	109.5
C5—C4—C11	120.01 (13)	H38A—C38—H38B	109.5
C4—C5—C6	123.45 (14)	C35—C38—H38C	109.5
C4—C5—H5	118.3	H38A—C38—H38C	109.5
C6—C5—H5	118.3	H38B—C38—H38C	109.5
C5—C6—C1	114.88 (13)	C41—C39—C40	111.4 (2)
C5—C6—Rh1	125.91 (11)	C41—C39—C32	109.49 (18)
C1—C6—Rh1	119.20 (11)	C40—C39—C32	109.21 (16)
C10—C7—C9	106.95 (13)	C41—C39—C42	107.85 (19)
C10—C7—C8	107.42 (14)	C40—C39—C42	107.2 (2)
C9—C7—C8	109.92 (14)	C32—C39—C42	111.70 (18)
C10—C7—C2	111.85 (13)	C39—C40—H40A	109.5
C9—C7—C2	109.87 (13)	C39—C40—H40B	109.5
C8—C7—C2	110.72 (13)	H40A—C40—H40B	109.5
C7—C8—H8A	109.5	C39—C40—H40C	109.5
C7—C8—H8B	109.5	H40A—C40—H40C	109.5
H8A—C8—H8B	109.5	H40B—C40—H40C	109.5
C7—C8—H8C	109.5	C39—C41—H41A	109.5
H8A—C8—H8C	109.5	C39—C41—H41B	109.5
H8B—C8—H8C	109.5	H41A—C41—H41B	109.5
C7—C9—H9A	109.5	C39—C41—H41C	109.5
C7—C9—H9B	109.5	H41A—C41—H41C	109.5
H9A—C9—H9B	109.5	H41B—C41—H41C	109.5
C7—C9—H9C	109.5	C39—C42—H42A	109.5
H9A—C9—H9C	109.5	C39—C42—H42B	109.5
H9B—C9—H9C	109.5	H42A—C42—H42B	109.5
C7—C10—H10A	109.5	C39—C42—H42C	109.5
C7—C10—H10B	109.5	H42A—C42—H42C	109.5
H10A—C10—H10B	109.5	H42B—C42—H42C	109.5
C7—C10—H10C	109.5	C44—C43—C50	124.99 (16)
H10A—C10—H10C	109.5	C44—C43—Rh1	71.63 (9)
H10B—C10—H10C	109.5	C50—C43—Rh1	110.62 (11)
C13—C11—C14	109.46 (15)	C44—C43—H43	117.5
C13—C11—C12	108.31 (14)	C50—C43—H43	117.5
C14—C11—C12	107.39 (14)	Rh1—C43—H43	87.7
C13—C11—C4	108.40 (13)	C43—C44—C45	126.98 (17)
C14—C11—C4	110.58 (13)	C43—C44—Rh1	73.16 (9)
C12—C11—C4	112.65 (13)	C45—C44—Rh1	105.91 (11)
C11—C12—H12A	109.5	C43—C44—H44	116.5
C11—C12—H12B	109.5	C45—C44—H44	116.5
H12A—C12—H12B	109.5	Rh1—C44—H44	91.0
C11—C12—H12C	109.5	C44—C45—C46	114.42 (15)
H12A—C12—H12C	109.5	C44—C45—H45A	108.7
H12B—C12—H12C	109.5	C46—C45—H45A	108.7
C11—C13—H13A	109.5	C44—C45—H45B	108.7
C11—C13—H13B	109.5	C46—C45—H45B	108.7
H13A—C13—H13B	109.5	H45A—C45—H45B	107.6
C11—C13—H13C	109.5	C47—C46—C45	113.85 (14)
H13A—C13—H13C	109.5	C47—C46—H46A	108.8
H13B—C13—H13C	109.5	C45—C46—H46A	108.8
C11—C14—H14A	109.5	C47—C46—H46B	108.8
C11—C14—H14B	109.5	C45—C46—H46B	108.8
H14A—C14—H14B	109.5	H46A—C46—H46B	107.7
C11—C14—H14C	109.5	C48—C47—C46	125.49 (16)
H14A—C14—H14C	109.5	C48—C47—Rh1	70.95 (9)
H14B—C14—H14C	109.5	C46—C47—Rh1	110.94 (11)
C20—C15—C16	121.96 (13)	C48—C47—H47	117.3
C20—C15—O2	119.54 (13)	C46—C47—H47	117.3
C16—C15—O2	118.50 (13)	Rh1—C47—H47	88.1
C15—C16—C17	115.79 (13)	C47—C48—C49	126.68 (17)
C15—C16—C21	122.80 (13)	C47—C48—Rh1	72.85 (9)
C17—C16—C21	121.40 (13)	C49—C48—Rh1	107.18 (11)
C18—C17—C16	123.92 (14)	C47—C48—H48	116.7
C18—C17—H17	118.0	C49—C48—H48	116.7
C16—C17—H17	118.0	Rh1—C48—H48	90.0
C19—C18—C17	117.44 (14)	C48—C49—C50	114.31 (16)
C19—C18—C25	120.09 (14)	C48—C49—H49A	108.7
C17—C18—C25	122.44 (14)	C50—C49—H49A	108.7
C20—C19—C18	120.73 (14)	C48—C49—H49B	108.7
C20—C19—H19	119.6	C50—C49—H49B	108.7
C18—C19—H19	119.6	H49A—C49—H49B	107.6
C19—C20—C15	120.13 (14)	C43—C50—C49	113.05 (15)
C19—C20—H20	119.9	C43—C50—H50A	109.0
C15—C20—H20	119.9	C49—C50—H50A	109.0
C24—C21—C23	107.75 (14)	C43—C50—H50B	109.0
C24—C21—C22	107.24 (14)	C49—C50—H50B	109.0
C23—C21—C22	110.05 (14)	H50A—C50—H50B	107.8
C24—C21—C16	111.22 (13)	C1—O1—P1	113.24 (9)
C23—C21—C16	110.32 (13)	C15—O2—P1	125.89 (9)
C22—C21—C16	110.18 (13)	C29—O3—P1	120.58 (9)
C21—C22—H22A	109.5	O2—P1—O1	103.46 (6)
C21—C22—H22B	109.5	O2—P1—O3	98.37 (6)
H22A—C22—H22B	109.5	O1—P1—O3	100.63 (6)
C21—C22—H22C	109.5	O2—P1—Rh1	118.11 (4)
H22A—C22—H22C	109.5	O1—P1—Rh1	109.60 (4)
H22B—C22—H22C	109.5	O3—P1—Rh1	123.72 (4)
C21—C23—H23A	109.5	C6—Rh1—P1	79.14 (4)
C21—C23—H23B	109.5	C6—Rh1—C48	161.26 (6)
H23A—C23—H23B	109.5	P1—Rh1—C48	99.05 (5)
C21—C23—H23C	109.5	C6—Rh1—C47	162.53 (6)
H23A—C23—H23C	109.5	P1—Rh1—C47	100.73 (4)
H23B—C23—H23C	109.5	C48—Rh1—C47	36.20 (7)
C21—C24—H24A	109.5	C6—Rh1—C44	92.63 (6)
C21—C24—H24B	109.5	P1—Rh1—C44	156.94 (5)
H24A—C24—H24B	109.5	C48—Rh1—C44	95.45 (6)
C21—C24—H24C	109.5	C47—Rh1—C44	80.75 (6)
H24A—C24—H24C	109.5	C6—Rh1—C43	96.87 (6)
H24B—C24—H24C	109.5	P1—Rh1—C43	166.20 (5)
C27—C25—C18	112.33 (14)	C48—Rh1—C43	80.44 (6)
C27—C25—C28	108.38 (16)	C47—Rh1—C43	87.04 (6)
C18—C25—C28	110.19 (14)	C44—Rh1—C43	35.21 (6)
C27—C25—C26	108.72 (15)	C52A—C51A—C56A	120.0
C18—C25—C26	108.24 (13)	C52A—C51A—C57A	120.63 (19)
C28—C25—C26	108.92 (15)	C56A—C51A—C57A	119.37 (19)
C25—C26—H26A	109.5	C53A—C52A—C51A	120.0
C25—C26—H26B	109.5	C53A—C52A—H52A	120.0
H26A—C26—H26B	109.5	C51A—C52A—H52A	120.0
C25—C26—H26C	109.5	C54A—C53A—C52A	120.0
H26A—C26—H26C	109.5	C54A—C53A—H53A	120.0
H26B—C26—H26C	109.5	C52A—C53A—H53A	120.0
C25—C27—H27A	109.5	C53A—C54A—C55A	120.0
C25—C27—H27B	109.5	C53A—C54A—H54A	120.0
H27A—C27—H27B	109.5	C55A—C54A—H54A	120.0
C25—C27—H27C	109.5	C56A—C55A—C54A	120.0
H27A—C27—H27C	109.5	C56A—C55A—H55A	120.0
H27B—C27—H27C	109.5	C54A—C55A—H55A	120.0
C25—C28—H28A	109.5	C55A—C56A—C51A	120.0
C25—C28—H28B	109.5	C55A—C56A—H56A	120.0
H28A—C28—H28B	109.5	C51A—C56A—H56A	120.0
C25—C28—H28C	109.5	C51A—C57A—H57A	109.5
H28A—C28—H28C	109.5	C51A—C57A—H57B	109.5
H28B—C28—H28C	109.5	H57A—C57A—H57B	109.5
C34—C29—O3	119.18 (13)	C51A—C57A—H57C	109.5
C34—C29—C30	122.32 (14)	H57A—C57A—H57C	109.5
O3—C29—C30	118.49 (13)	H57B—C57A—H57C	109.5
C31—C30—C29	115.06 (14)	C52B—C51B—C56B	120.0
C31—C30—C35	121.96 (14)	C52B—C51B—C57B	119.97 (12)
C29—C30—C35	122.98 (14)	C56B—C51B—C57B	120.01 (12)
C30—C31—C32	124.22 (15)	C51B—C52B—C53B	120.0
C30—C31—H31	117.9	C51B—C52B—H52B	120.0
C32—C31—H31	117.9	C53B—C52B—H52B	120.0
C33—C32—C31	117.71 (15)	C52B—C53B—C54B	120.0
C33—C32—C39	121.97 (17)	C52B—C53B—H53B	120.0
C31—C32—C39	120.31 (17)	C54B—C53B—H53B	120.0
C32—C33—C34	120.43 (16)	C55B—C54B—C53B	120.0
C32—C33—H33	119.8	C55B—C54B—H54B	120.0
C34—C33—H33	119.8	C53B—C54B—H54B	120.0
C29—C34—C33	120.19 (15)	C54B—C55B—C56B	120.0
C29—C34—H34	119.9	C54B—C55B—H55B	120.0
C33—C34—H34	119.9	C56B—C55B—H55B	120.0
C30—C35—C38	111.24 (15)	C55B—C56B—C51B	120.0
C30—C35—C36	110.91 (13)	C55B—C56B—H56B	120.0
C38—C35—C36	107.48 (15)	C51B—C56B—H56B	120.0
C30—C35—C37	109.89 (13)	C51B—C57B—H57D	109.5
C38—C35—C37	106.99 (14)	C51B—C57B—H57E	109.5
C36—C35—C37	110.24 (16)	H57D—C57B—H57E	109.5
C35—C36—H36A	109.5	C51B—C57B—H57F	109.5
C35—C36—H36B	109.5	H57D—C57B—H57F	109.5
H36A—C36—H36B	109.5	H57E—C57B—H57F	109.5
